# Love-Lights

**Published:** 1888-02-18

**Authors:** J. A. Thomson

**Affiliations:** Lecturer on Zoology in the University of Edinburgh, and Joint-Editor of the *London Microscopical Journal*


### Love-Lights.

By J. A. Thomson, M.A., Lecturer on Zoology in the University of Edinburgh, and Joint-Editor of the *London Microscopical Journal.*

Both among plants and animals the advent of love frequently transfigures the life. Are not the petals of flowers, according to Mr. Ruskin, their marriage robes? Do not even the cold-blooded fishes sometimes blush with love, and does not the male newt—apt to be thought somewhat unemotional—become quite a beau? And what shall we say of the many birds which so ostentatiously go a-courting, with love glowing in endless variety of colour and ornament. But we are not going to talk of these; we must have something more sensational. It is well known that not a few animals live so brightly that they glow. Their souls seem to shine through their bodies—in other words, they are phosphorescent. We don't know very much about this, and do not propose discussing it now. It is enough to recall the fact that in some cases the flame of life burns very brightly indeed, and that these animals shine. In the Mexican Cuenyos, it was recently shown that the rapid burning is almost entirely dependent on the rate of breathing. By artificially blowing air into the breathing tubes, the insects were made to blaze.

Now it has been long known that love is also a flame. For this statement there are abundant authorities. But the fact is nowhere better illustrated than in the glowworm beetles. We need not repeat the familiar story of how the wingless female attracts her winged mate by a warm light, which seems to be greater than that of which he can boast. We wish rather to tell the love story of another species, common in Southern Europe—the Italian Lucciola. It is not always that a general reader of the bulletins of the Italian Entomological Society falls in with such a gem of a paper as that in which Professor Emery not long ago described his eavesdropping on Lucciola. The Birds of Paradise, Mr. Wallace tells us, become so pre-occupied with their courtships, or their "dancing parties," as the natives call them, that they will allow the crowd of rivals to be very considerably thinned by arrows before they will desist from their flirtations. So those Italian glowworms were not at all shamed by the presence of "a chiel amang them takin' notes." They were not at all embarrassed in their wooing.

It must be a beautiful sight to see these living flashes of light gradually appearing from their hiding-places as the shades deepen, and illumining copse and meadow with their torches of love. The flying Lucciolas do not seem to feed at all; they live on the stores which they have laid up for their honeymoon. What is slowly accumulated in their bodies during larval life is consumed with prodigal rapidity in the feverish ecstasy of their few days of love. They literally waste their substance in riotous living. It seems probable enough that their brilliancy is also of use to lighten their path, as it appeared to brighten when the way was difficult and full of obstacles; or perhaps these brightenings were simply flashes of temper. But there can be no doubt that in the main the lights are love's beacons. Between nine and ten the males may be seen flying restlessly about, searching with their large eyes for their mates. They, "little coquettes" (civettuole), as the Professor calls them, are lying waiting among the grass, and when they see the passing flash send forth tremulous timid signals. "Then begins love's duet, where the flashes of light take the place of trills and quavers." He might have said kisses when he was at it, were such a term not beneath the dignity of a grave scientist. The lover begins to dance with delight round his mistress, shining most brilliantly all the time. There is not much difference in their respective flashes; the colour of light is the same in both, the intensity appears equal, but the brightness of the female appears over a more restricted area. The signals of the male are shorter, follow one another more rapidly, and are stronger while they last. No insinuations, sir! Those of the female are longer, follow one another more slowly, and have something tremulous about them, especially when waning. What marvellous unity there is in nature. I am only referring, fair reader, to the constancy to be observed in the perigenesis of the plastidule. To return to the love story, Professor Emery was wicked enough to shut up females in glass tubes, and to watch them sending impotent signals through their prison walls. There's no limit to what a man may do. He even tried to take a number home that he might have the pleasure of watching them without sitting in the meadow till midnight, but they resented this and would not work.

Those females are rather heartless after all, most sinfully fond of flirtation. For no sooner have they entangled one admirer than they proceed to attract another, and another, and another; and so little circles of coquetry are formed, the mistress in the centre blazing up into brilliant attractiveness if yet another should pass that way, the suitors all round her dancing and flaring like little demons, or, after a while, getting tired and sulky. What a world we live in! It is not my business to inquire whether we have improved on the habits of the Lucciola. We need diamonds to make us shine, at any rate. The "phalanx of adorers" seems to lose its temper occasionally and comes to blows, and one cannot wonder; the centre of attraction, however, preserves her equanimity wonderfully, and seems only interested in increasing the number of suitors. Professor Emery watched many a circle, subsiding into quietness after a while, but still bewitched. As midnight came the living lights became scarcer, the flying searchers sought their homes unmated, the charmed circles sat on: for many it was only another case of "love's labour lost."

